# Classification of Alzheimer’s Disease with and without Imagery Using Gradient Boosted Machines and ResNet-50

**DOI:** 10.3390/brainsci9090212

**Published:** 2019-08-22

**Authors:** Lawrence V. Fulton, Diane Dolezel, Jordan Harrop, Yan Yan, Christopher P. Fulton

**Affiliations:** 1 Department of Health Administration, Texas State University, 601 University Drive, San Marcos, TX 78666, USA; 2 Acushnet Holdings Corporation, Acushnet, MA 02743, USA; 3 United States Air Force Experimental Test Pilot School, Edwards Air Force Base, CA 93524, USA

**Keywords:** Alzheimer’s disease, extreme gradient boosting, deep residual learning, convolutional neural networks, machine learning, dementia

## Abstract

Background. Alzheimer’s is a disease for which there is no cure. Diagnosing Alzheimer’s disease (AD) early facilitates family planning and cost control. The purpose of this study is to predict the presence of AD using socio-demographic, clinical, and magnetic resonance imaging (MRI) data. Early detection of AD enables family planning and may reduce costs by delaying long-term care. Accurate, non-imagery methods also reduce patient costs. The Open Access Series of Imaging Studies (OASIS-1) cross-sectional MRI data were analyzed. A gradient boosted machine (GBM) predicted the presence of AD as a function of gender, age, education, socioeconomic status (SES), and a mini-mental state exam (MMSE). A residual network with 50 layers (ResNet-50) predicted the clinical dementia rating (CDR) presence and severity from MRI’s (multi-class classification). The GBM achieved a mean 91.3% prediction accuracy (10-fold stratified cross validation) for dichotomous CDR using socio-demographic and MMSE variables. MMSE was the most important feature. ResNet-50 using image generation techniques based on an 80% training set resulted in 98.99% three class prediction accuracy on 4139 images (20% validation set) at Epoch 133 and nearly perfect multi-class predication accuracy on the training set (99.34%). Machine learning methods classify AD with high accuracy. GBM models may help provide initial detection based on non-imagery analysis, while ResNet-50 network models might help identify AD patients automatically prior to provider review.

## 1. Introduction

Alzheimer’s disease (AD) is a degenerative brain disease with no cure [1]. AD is the most common type of dementia; it is the sixth leading cause of death in the United States [2]. AD is characterized by progressive cerebral cortex atrophy leading to memory loss, increasing cognitive deficits, and potential loss of motor functions [3]. In 2019, there were 5.8 million people of all ages diagnosed with AD, the prediction is 14 million with AD by 2050 [2]. Specifically, one in ten people over 65 years of age have AD, with females more likely to have AD than males [3].

Due to the lengthy prodromal period for AD, it is commonly diagnosed in seniors [4]. Earlier diagnosis of AD would facilitate treatment interventions and family planning, and it could save up to $7.9 trillion dollars annually [2]. Unfortunately, differential diagnosis of AD is an intense, time consuming, and costly process involving physical and mental exams, laboratory and neurology tests, as well as neurological imaging (magnetic resonance imaging—MRI, computed tomography—CT, and positron emission tomography—PET) [4]. As an example, the cost for a brain MRI may range from $600 to $1300 [5], and for the uninsured or underinsured this might be infeasible.

### 1.1. Background

Dementia is not identical to the normal mental deterioration associated with aging [2]. It is a progressive, chronic disease with symptoms that may include the loss of memory, communication problems, behavioral changes, and personality changes. Individuals with dementia are often not oriented to time or place and may not remember to eat regularly or practice good hygiene. AD is the most common (60%–80%) form of dementia [2]. Normal “forgetful” elderly individuals can be assisted to remember; however, those with AD may not even recall reminders. This fact is unsurprising since the etiology of AD (and other dementias) is damage to brain cells visible by imagery. AD is associated with atrophy of the cerebral cortex, which is the outer (grey wrinkled) covering of the cerebrum in the brain. The irreversible atrophy derives from amyloid plaque formation and neurofibrillary tangles. Amyloid (protein) plaques bind to and destroy nerve synapses (where nerves touch) resulting in memory loss, an early symptom of AD. Neurofibrillary tangles, from a twisted protein strand, damage neurons, and nerve synapses.

AD has an extremely slow onset. The prodromal period may be 10–20 years while plaques and tangles continue spreading through the cortex [3]. Definitive diagnosis is complicated because amyloid plaque formation and neurofibrillary tangles are present even in seniors with good cognitive functioning [6,7], although in lesser numbers and without the predictable pattern present in AD brain scans. To that end, diagnostics from brain MRIs, CT scans, and PET scans are useful for monitoring the progression of AD. Brain volume atrophy, especially in the hippocampal areas, also reflects the decline in cognitive functions characteristic of AD pathology [8].

Scores from longitudinal clinical tests, like the mini-mental state exam (MMSE), clinical dementia rating (CDR), and Alzheimer’s disease assessment scale (ADAS), have shown high correlation with AD disease progression [4,9]. Mapping this association is helpful because it allows clinicians to provide interventions in the asymptomatic preclinical period. To clarify, while there is no cure for AD the progression may be slowed, and the symptoms treated. Common treatments include memory drugs, behavior mediation, sleep treatments, occupational therapy, and assisted living [2]. Thus, the early identification of AD is vital to slowing the progression of the disease.

### 1.2. Related Research

Mapping the progression of AD thorough all six stages requires complex non-linear multifactorial multi-modal modeling. Researchers have used growth mixture modeling (GMM) to model the decay of preclinical MMSE scores of individuals (*n* = 528) over 75 years of age [10]. GMM was used to detect both group and individual changes. Bhagwat et al. [4] modeled AD symptom progression classes for one thousand participants over six years with the MMSE scores and the AD AS scale. The authors predicted AD progression using MRI imaging, genetic and clinical characteristics, and by applying a Siamese neural network, which is a network with two or more identical equally weighted subnetworks. Zhang et al. [11,12] used a subset of the Open Access Series of Imaging Studies (OASIS) data (*n* = 126) and proposed methods to identify binary-coded AD using Eigenbrain imagery. They eliminated individuals under 60 and incomplete observations, modeled AD as binary, limited their analyses to a very small subset of coronal slice images, and used 10-fold cross-validation run 50 times. (Our approach is somewhat similar; however, in this paper, we use 51 slices, all available imagery data, random image generation, and multi-class classification using ResNet-50 on a 20% blinded validation set.) Bansal et al. [13] applied several machine learning (ML) models to the OASIS brains dataset to classify binary-coded AD; however, they used the entirety of the data set, which may have resulted in overfitting and prevented the generation of true performance metrics.

A related study focused on predicting the individual Alzheimer’s disease assessment scale (ADAS) and MMSE scores [14]. The researchers used an anatomically partitioned artificial neural network, cross-sectional baseline data sets, three sets of Alzheimer’s disease neuroimaging initiative cohort data, and two MRI measures. Another study used MRI brain images of tissue perfusion and perfusion scores to develop discrimination maps, which successfully distinguished AD, mild cognitive decline, and subjective cognitive decline [15]. Other researchers applied ML to Alzheimer’s disease neuroimaging initiative data (*n* = 805) to predict AD clinical scores [9]. By using soft-split sparse regression-based random forests to estimate missing longitudinal scores they produced more accurate predictions than traditional random forest methods.

Other research demonstrated that fractal descriptors were more accurate for support vector machine (SVM) classification of abnormal MRI brain images for AD than discrete wavelet transform or empirical mode decomposition [16]. The authors achieved at best 87% binary class classification accuracy on the OASIS-1 dataset using variational mode decomposition. In a very small sample, another study on automatic classification of AD achieved 100% accuracy using SVM and 10-fold cross-validation with 35 test images and 35 controls [17]. Another study used 33 T2-weighted, MRI brain images and used a multiclass SVM, which correctly classified 91% of the 33 images after training [18]. Due to the small size of images, none of the models discussed were applied to a blinded validation set. The study also considered cognitive scores but did not build models devoid of imagery data to estimate performance. Further, neither of these studies provides a multi-class classification nor evaluates non-imagery models as done in this study.

This work extends these researchers’ efforts. This study has two objectives. The first objective is to build machine learning models, specifically, gradient boosted machines (GBM), that effectively predict both the presence of AD using ML methods without imagery or imagery-related variables. The second objective is to build imagery models using deep learning techniques, specifically a residual network with 50 layers (ResNet-50), that improves previous classification efforts. The significance of this paper follows. 

1. Our multi-class classification of 98.99% may be the highest in the literature for multi-class classification, particularly on a blinded validation set.

2. There are no other readily available papers that apply ResNet-50 to MRI AD detection.

3. This may be the first paper to compare both imagery and non-imagery analysis. 

Non-imagery methods may be particularly relevant to the poor and underserved, as methods that adequately forecast AD without imagery reduce the costs of imaging. Imagery methods that are increasingly accurate may help providers with diagnostics.

## 2. Materials and Methods

### 2.1. Data and Software

The Open Access Series of Imaging Studies (OASIS-1) is publicly available data that provides researchers access to cross-sectional and longitudinal MRI data [19]. The data derive from the work of Marcus et al. [20]. The full data set contains cross-sectional data (*N =* 416) derived from MRI studies at Washington University. The OASIS-1 data include MRI imagery in image format as well as patient socio-demographics and clinical variables in data frame format. In this study, we used the atlas-registered, gain-field corrected images provided as well as the associated data in the data frame. Many studies use the Alzheimer’s disease neuroimaging initiative (for examples, see [14,15]), and many others use OASIS-1 (for examples, see [11,12]). For our study, OASIS-1 is readily accessible, provides reasonable demographics for proof of concept, and extends well longitudinally for future work.

The MRI imagery data included {x = 176, y = 208, z = 176} images of the {sagittal (x,y), axial (x,z), coronal (y,z)} planes for each of the *N* = 416 observations, respectively. The second dimension, y = 208, was defined as the number of slices. All images were from right-handed men and women (a control) aged 18 to 96. Both the imagery and non-imagery data sources were used in this study. In this study, the imagery data were restricted to the center 51 slices (78 through 128), as the outer slices contain less information about AD presence and severity. The total number of valid training images used for classification was 16,661 using and 80% training set; however, images were processed through an image generation algorithm to increase the learning of the ResNet model. The remaining 4139 images were used as a validation set. Images were padded with 3 voxels, so the total image voxels used was over 644 million.

Both R Statistical Software version 3.5 (“Feather Spray”) [21] and Anaconda Python version 3.6 [22] were used for this study. R was used for processing the labels and non-imagery analysis, while Python was used for constructing and running the ResNet-50 model.

### 2.2. Dependent Variable

For this study, the single response variable (label) was the Washington University Clinical Dementia Rating (CDR), which was developed to measure dementia severity [7]. The CDR was measured as follows:{0= non-demented; 0.5 = very mild dementia; 1 = mild dementia; 2 = moderate dementia}. This instrument is reliable in estimating clinical dementia, which is largely related to AD [2]. Since only two cases in the *N* = 416 data set involved moderate dementia, the values of CDR were recoded as follows: {0 = non-demented, 1 = very mild dementia, 2 = mild or moderate dementia}. This recoding still resulted in imbalanced classes. The distribution of the dependent variable values was {316, 70, 30} for {no dementia, very mild dementia, mild or moderate dementia}, respectively.

There were also three sets of predictor variables available in the OASIS-1 dataset: Demographic variables, clinical predictor variables, and imagery predictor variables. Each of these sets of variables is defined in the following sections.

#### 2.2.1. Socio-Demographic Predictor Variables, Non-Imagery Data

Several independent variables from the OASIS-1 dataset provided patient sociodemographic characteristics. A listing of these variables with their associated operational definitions follows.

- Gender: {0 = Female, 1 = Male}, 100% complete (416 of 416);
- Age: (18, 96) years of age, 100% complete (416 of 416);
- Education: {1 < high school (HS), 2 = HS Graduate, 3 = Some College, 4 = College Graduate, 5 = Beyond College Graduate}, 56% complete (235 of 416);
- Socioeconomic Status (SES): (1 = lower, 2 = lower middle, 3 = middle, 4 = upper middle, 5 = upper), 52% complete (216 of 416).

While much of the data were complete, education and SES were not. Imputation using the median was used to facilitate the non-imagery analysis. While a large percentage of observations were missing, analyses were run omitting the variables, including non-response as a factor level, and including imputation using the median. The median was selected based on performance.

#### 2.2.2. Clinical Predictor Variables, Non-Imagery Data

Clinical predictor variables were also available. These variables include the mini-mental state exam (MMSE) score, the atlas scaling factor, the estimated total intracranial volume, and the normalized whole brain volume. Three of the four variables require imagery (all except the MMSE), so they were not used in the non-imagery model. The definitions of these variables follow.

- Mini-mental state exam (MMSE): (0,30). The MMSE is a 30-point questionnaire that has been shown to be valid and reliable in identifying dementia [7,23]. The variable was 56% complete (235 of 416). Missing values were imputed with the median. The MMSE was included in the non-imagery model.
- Atlas scaling factor (ASF): (0.88–1.56) (observed). The ASF is a one-parameter scaling factor that allows for comparison of the estimated total intracranial volume (eTIV) based on differences in human anatomy [19]. This variable was 100% complete (416 of 416) [10].
- Estimated total intracranial volume (eTIV): (1132–1992) mm^3^ [24]. The eTIV variable estimates intracranial brain volume. This variable was 100% complete (416 of 416).
- Normalized whole brain volume (nWBV): (0.64–0.90) mg (observed). This variable measures the volume of the whole brain. This variable was 100% complete (416 of 416).

#### 2.2.3. Imagery Variables

**Figure 1:**
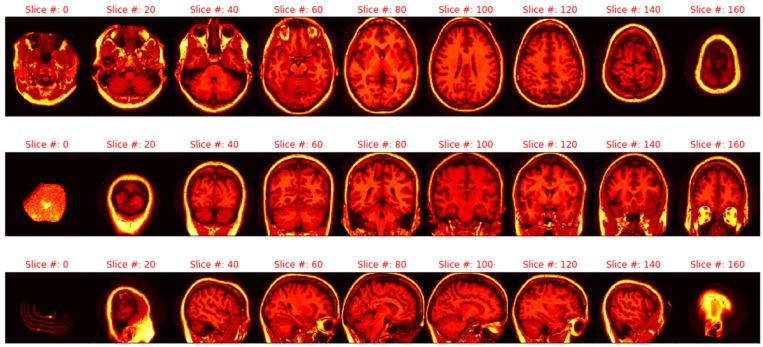
Axial, sagittal, and coronal slices (respectively).

The imagery used in this study was the masked version of the gain-field corrected, atlas-registered image to the Talairach atlas space. These images were preprocessed with spatial normalization of the 3D brain images. The intensity inhomogeneity (IIH) or gain field are intensity variations (i.e., noise) not related to the patient’s anatomical image, which include patient movement, nearby static fields, radio-frequency variation, or other non-patient sources [25]. These gain field variations were corrected to form an averaged 3D image. Next, the identifying 3D facial and non-brain values were masked. The Talairach space is, then, a 3D coordinate system (called an atlas) used for mapping locations of brain features [26]. The averaged 3D images are spatially normalized into the Talairach space with a transform that maps the subject’s brain image to a reference brain and smooths out individual brain anatomy size and shape differences [26,27]. Figure 1 illustrates the sagittal, axial, and coronal viewpoints as well as the resolution used in this study.

### 2.3. Non-Imagery Models 

#### 2.3.1. Imputation, Cross Validation, and Pseudo-Random Seeds

For the non-imagery data file, imputation was used to complete missing data fields. Education level and socio-economic status (SES) had high percentages of missing data (44% and 48%, respectively). Medians were imputed to investigate whether the variables might still be useful in forecasting. All other variables were 100% complete, including all imagery.

K-fold cross validation was used to assess the non-imagery model, where *k* = 10. This technique subdivides the data into five separate folds or sections. Nine folds are used for training the models, and one-fold is used for evaluating the performance after training. Each fold serves as a validation set one time, and performance metrics are calculated based on the results from all 10 training runs.

Pseudo-random seeds were used for the replication of the results. The use of random seeds ensures that the results were not due simply to the selection of random numbers via pseudo-random number generation. Further, random seeds allow for the replication of exact results. 

#### 2.3.2. Gradient Boosted Tree Ensembles (Gradient Boosting)

Gradient boosted tree ensembles were used to classify AD using the socio-demographic variables and MMSE. A gradient boosted tree ensemble, known often as gradient boosted machines (GBM), is a model that optimizes prediction accuracy based on iterations of weaker decision/classification tree models. A classification tree model seeks to split the independent variables at various points in an effort to create a decision tree that provides a classification vote. Often, the Gini impurity or cross-entropy formulae are used to determine those splits (Equation 1 and Equation 2) [28,29]. In these equations, *k* is the number of classes and *p_i_* is the proportion of cases belonging to case *i*.

(1) $$\mathrm{Gini}\;\mathrm{impurity}:\;\sum_{i=1}^{k}p_{i}(1-p_{i}).$$

(2) $$\mathrm{Cross}-\mathrm{entropy}:\;–\sum_{i=1}^{k}p_{i}\log\left(p_{i}\right)$$

**Figure 2:**
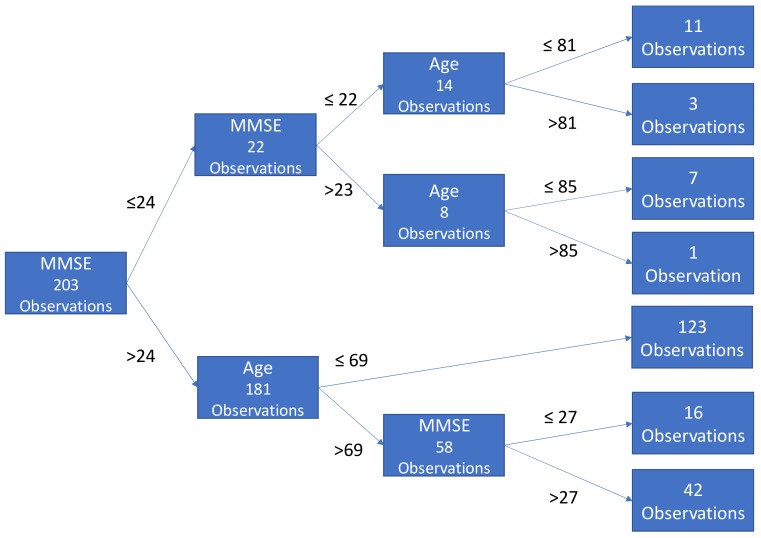
A sample tree classification with a maximum of three branches. MMSE - mini-mental state exam.

With these equations, in a greedy fashion the splitting algorithm is trying to achieve homogeneity within each subset of the tree. To avoid overfitting, the trees are pruned by limiting the number of internal splits/branches. In this implementation, individual trees grew no more than three branches to avoid overfitting. Each tree recommends a classification status (e.g., no AD or AD). Figure 2 illustrates a sample classification tree.

At a high level, a gradient boosted tree ensemble is a collection of tree models that is derived by sequentially incorporating, in an additive manner, direct residual errors (i.e., boosting). At each iteration a differentiable loss function is optimized (e.g., area under the curve) [30]. A good discussion of trees, forests, and boosting is from Chen [31].

The R Statistical Software Package, XGBoost [32], was used to implement gradient boosting. The XGBoost implementation of gradient boosting is ideal for this use case. First, this approach has seen considerable success in prediction accuracy across a variety of datasets. Second, XGBoost takes advantage of unique algorithms that minimizes computation complexity, which allows gradient boosting to scale fairly easily in parallel fashion. A hyperparameter grid search provided parameters for use in building the trees and a ten-fold cross-validation provided accuracy metrics.

### 2.4. Imagery Data

#### 2.4.1. Pre-processing: Min–Max Scaling, PCA Investigation, and Pseudo-Random Seeds

While gradient-boosted forests are scale invariant, ResNet-50 (an adaptation of a CNN) is not. The data for the ResNet were min–max scaled based on the data set minimum and maximum values, {0, 5089}. The minimum value for any voxel is known to be zero; however, extracting the maximum value ensured that no values in either the training or validation set would be below zero. The leakage of information from the validation set was at most one value (assuming 5089 was randomly assigned to the validation set).

After splitting the data into training and validation sets, principal component analysis (PCA) was used to extract significant Eigenbrain imagery separately to avoid bias [33,34]. Eigenbrains are reconstructed images based on Eigenvectors (orthogonal). Analysis of these structures, rather than original imagery, failed to improve the performance of the ResNet. The NiBabel library in Anaconda Python 3.6 provided the MRI import utility, while scikit-learn provided the PCA algorithm. Once again, pseudo-random seeds were used for replication in all analysis.

#### 2.4.2. Extraction and Manipulation of Individual Images

The 416 observations contained 208 slices of 176 voxels × 176 voxels. Fifty-one of these slices (range 78–128) were used in the analysis. These slices were extracted and saved as .png single channel (grayscale) files from the original format in a separate directory. Matching classification labels were replicated for each individual picture (i.e., CDR = 0 for patient 1 was replicated 51 times for each associated slice.) As part of the modeling process, the images were padded with 3 voxels on all sides and subjected to random image transformation to improve the ML learning. Not all images were valid. Instead of 51 × 416 = 21,216 images, there were only 20,800 contained within all MRIs. Without considering the unique (and infinite) transformations, the total images include more than 640 million voxels.

#### 2.4.3. Flow from the Dataframe, Training and Validation Set, and Training Image Generation

Due to the large size of the dataset, file names and associated labels for each slice were saved in a data frame. This data frame was randomly divided into two separate data frames, an 80% training set and a 20% validation set. A flow_from_dataframe function was used to load the image in batches of 128 (investigated through tuning) by randomly selecting file names in the training data frame. A training image generator then rescaled the data to be between 0 and 1 (dividing by 255 for grayscale). That same generator randomly rotated the images up to 20 degrees, zoomed up to 10%, shifted the height and width up to 10%, sheared up to 10%, and horizontally flipped some images. By editing the training images in this fashion, the ML model learns to adapt to deviations in the expected format. The validation data were left intact. No processing of those images was done to avoid the infusion of bias from manipulation. Those images were unknown.

#### 2.4.4. Deep Learning with Residual Networks (ResNet-50), and Imagery Data

ResNet-50 was used to classify CDR based solely on MRI imagery data. The model relied on Keras (TensorFlow backend) [35]. ResNet-50 is a residual deep learning network (with 50 layers), which attempts to address the problem of vanishing gradients that occur during back-propagation of convolutional neural networks (CNN). The ResNet-50 model was developed by He, Zhang, and Sun, and an ensemble of these ResNet models of various depths won imagery classification awards [36]. Increasing the depth of the network should increase the accuracy of the network, as long as over-fitting is considered. However, the problem with increased depth is that the signal required to change the weights, which arises from the end of the network by comparing ground-truth and prediction (observed versus predicted) becomes very small at the earlier layers because of the increased depth of the network. It essentially means that earlier layers remain almost unlearned. This is called the “vanishing gradient” problem, as the matrix of second order derivatives (the gradient) in the nonlinear optimization that attempts to tune weights becomes near zero. The second problem with training the deeper networks is, performing the optimization on huge parameter space and therefore naively adding the layers leading to higher training error. Residual networks allow training of such deep networks by constructing the network through modules called residual models. This is called the degradation problem. The ResNet-50 architecture is documented in [37]. The loss-metric optimized was categorical cross-entropy (formula 2).

**Figure 3:**
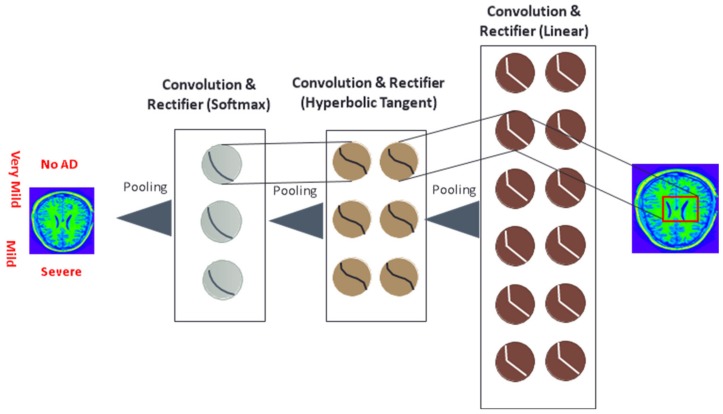
Example convolutional neural network for AD classification (read from right to left)

Convolutional blocks are a major component of the ResNet-50 model. These networks apply multiple filters (e.g., 3 × 3-pixel size filter) to images to classify. These filters are moved by strides across the original image. The values in the filters (which are learned) are multiplied against the values in the images. The results of those filters are pooled (e.g., maximum values extracted after the application of filters), which is effectively down sampling while retaining the most important features. Figure 3 is a symbolic picture of CNN.

## 3. Results

### 3.1. Descriptive Statistics

The data from the OASIS-1 study included 416 patient diagnostic files with 100 of those files confirming dementia. No patients under the age of 60 were diagnosed with dementia, as this is a rare event. One might assume that in GBM models, then, age would be the most important predictor (it is not). The table of dementia vs. non-dementia patients and associated statistics and frequencies adopted from the OASIS (2018) data is shown in Table 1. Due to the apparently dichotomous split at age 60, imagery and non-imagery models were run with the entirety of the dataset as well as with a subset of those 60 and above. ResNet-50 is capable of handling imbalanced data without techniques such as synthetic minority oversampling when trained sufficiently. Oversampling techniques often helps GBM models.

Most of the patients in the dataset exhibited no dementia (*n* = 316). Only two demonstrated moderate dementia. Table 2 provides the frequency distribution for CDR by gender. Due to only two individuals experiencing moderate dementia, CDR 1 and CDR 2 were combined for the imagery analysis.

The average patient in the dataset was 52.7 years old with a slightly less than perfect mental state evaluation (27.5 out of 30), an estimated brain volume of 1480.53 mm^3^, and 79% of the intracranial cavity occupied by the brain (nWBV). Table 3 displays statistics for the quantitative variable statistics.

**Figure 4:**
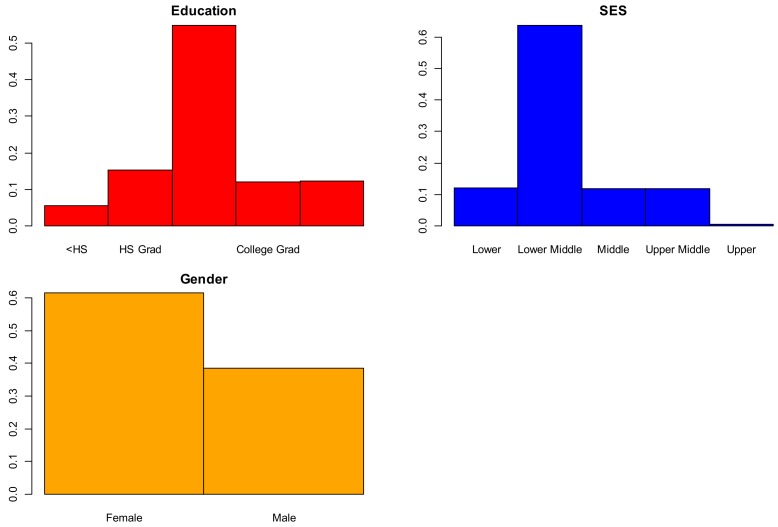
Bar charts of qualitative variables.

Qualitative variables model responses follow: 62% women, 28% college graduates, and 30% middle class. Spearman’s rank correlation between SES and education indicated a negative correlation (*r* = –0.715, *p* < 0.01). For the 121 individuals without a high school education, 119 were in the lower middle and lower classes. Figure 4 displays barplots for the variables of gender, SES, and education.

### 3.2. Correlations and Variable Transformations

**Figure 5:**
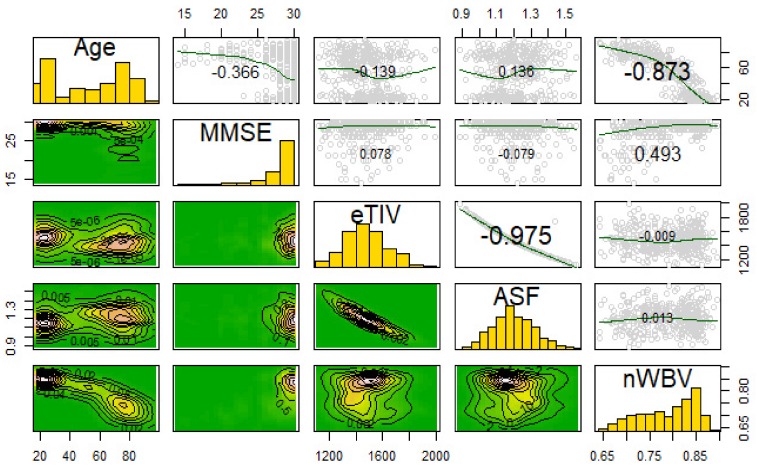
Correlation plots of all quantitative variables eTIV—estimated Total Intracranial Volume; ASF - Atlas Scaling Factor; nWBV—normalized Whole-Brain Volume.

Correlation between ASF and eTIV is negative and significant (t_414_ = −88.7, *p* < 0.001) as is the correlation between age and nWBV (t_414_ = −36.4, *p* < 0.001), an expected finding. Older individuals tend to lose brain volume. While some machine learning methods require transformations, tree methods are location/scale invariant, and ResNet (like all neural-net based models) benefits primarily from scaling. Thus, transformations (other than regularization through min–max scaling for imagery models) were not pursued. The scatterplot matrix of all variables is shown in Figure 5.

### 3.3. Transformation of MRIs and Eigenbrain Development

**Figure 6:**
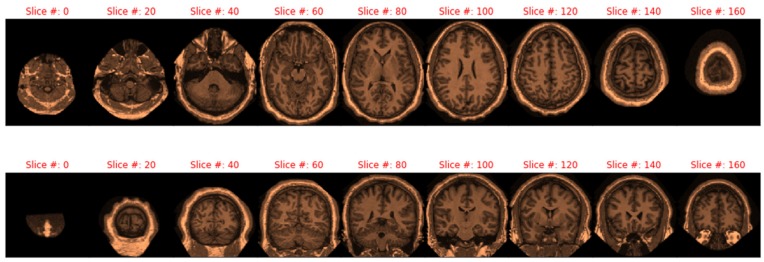

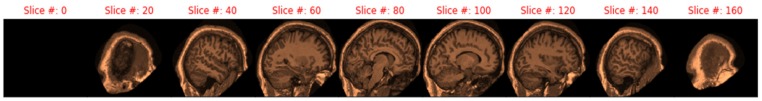
Eigenbrain imagery associated with Figure 1.

Figure 6 shows the Eigenbrain associated with Figure 1. The Eigenbrain images were derived via PCA. In PCA, images were restructured by building linear combinations of the original imagery where the first reconstructed image captures the most variability, the second captures the second most, etc. PCA maximizes the likelihood function in Equation 3 where Vector *a* and scalar *λ* form the Eigenspace (eigenvector and eigenvalue), while matrix *R* is the correlation matrix [38]. For each eigenvector indexed *i*, variance capture of the remaining variance was maximized. There are *k* total eigenvectors, where *k* is the number of columns in the dataset.

*Max*_*a*(*i*)_*L*_*i*_ = *a*^*T*^_*i*_*Ra*_*i*_ − λ (*a*^*T*^_*i*_*a*_*i*_), *I* = {1, 2…*k*}. (3)

### 3.4. Gradient Boosting with XGBoost and No Imagery

**Figure 7:**
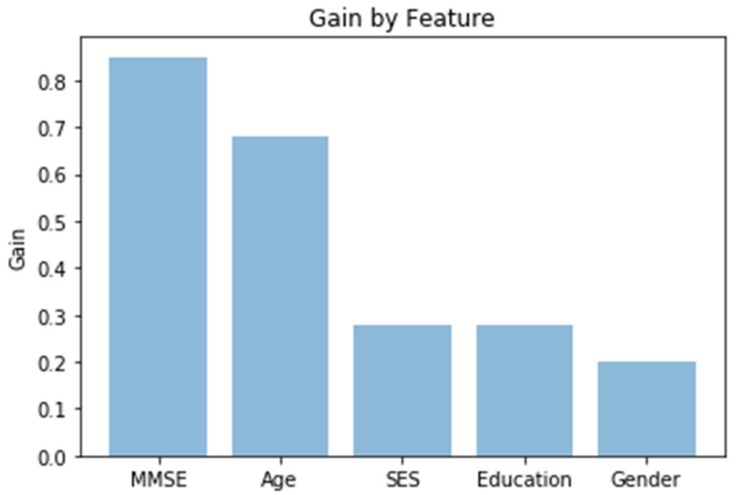
Variable importance. SES - socioeconomic status.

**Figure 8:**
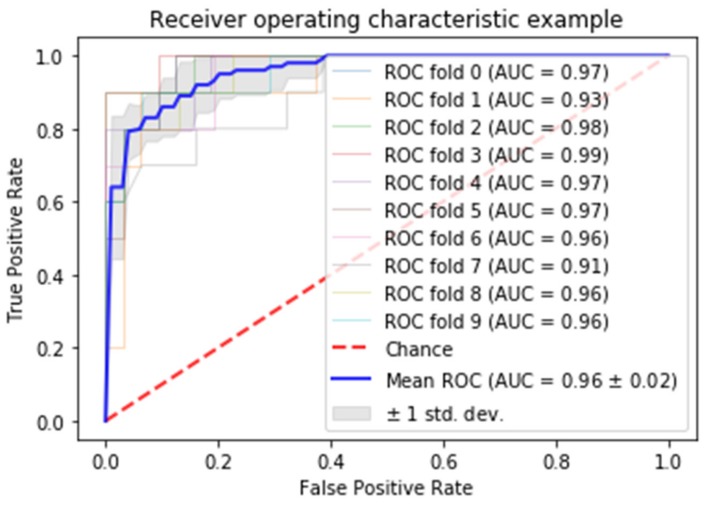
Receiver operating characteristic (ROC) curve. AUC—area under the curve.

Gradient boosted random forests were used to model dichotomous CDR (presence or absence) as a function of socio-demographics and MMSE scores (no imagery or imagery-derived variables such as ASF or eTIV). Hyperparameter grid search was used for tuning parameters. Ten-fold stratified cross-validation provided accuracy metrics. Stratification addresses the class imbalance problem. To avoid overfitting, the tree depth was constrained to three, a size investigated during grid search. The GBM achieved a mean of 91.3% classification accuracy with socio-demographic and MMSE variables. The gain, relative importance of the variable based on the appearance in the trees, is depicted in Figure 7, while Figure 8 is the receiver operating characteristic (ROC) curve. From Figure 7, the MMSE test would appear to be the most valuable classifier with age following. From Figure 8, the area under the curve is shown to be solidly in the 90th percentile for all of the 10 folds.

### 3.5. ResNet-50 (Deep Learning) with Keras

ResNet-50 was run to classify MRI imagery. The batch size for analysis was 64. Each epoch took between 213 and 711 seconds to run on an Acer Predator G-9 with an Intel I7-6700, 64GB of fast random-access memory, an NVidia GTX 980M graphical processing unit (GPU), running Windows 10. The maximum run time was due to additional processing activities. A total of 152 epochs were run, and the total run time for this analysis alone was 10.2 hours. The ResNet-50 was able to run on the computer’s GPU, although other processing functions were off-loaded to the central processing unit (CPU). Initial runs on the GPU were at least four times as long.

**Figure 9:**
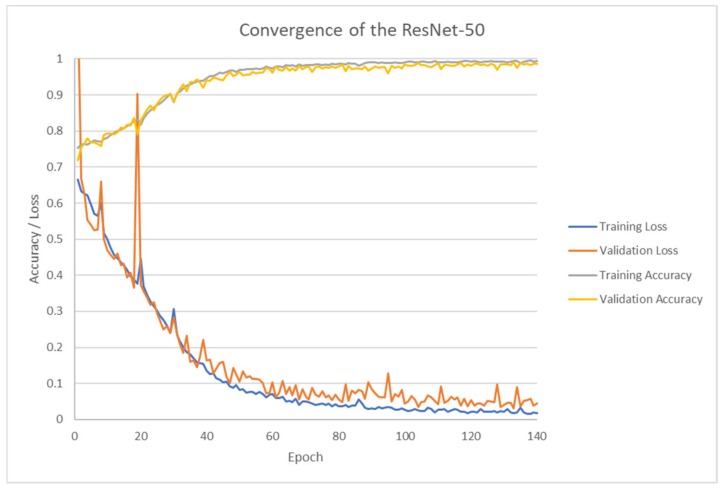
Classification accuracy versus epoch.

As depicted in Figure 9, ResNet-50 performed on the training set correctly (and surprisingly) classified 98.99% of the validation set at 133 epochs. The training set and test set nearly converged in accuracy. The loss metrics dropped accordingly to near zero on both sets. ResNet-50 proves to be a good automatic classifier. 

## 4. Discussion

Extreme gradient-boosted tree models provided 91.3% accuracy with only cognitive and socio-demographics data. The two most important variables in predicting AD were MMSE and (of course) age. What is interesting is that MMSE had higher feature importance when no patients below 60 in the dataset had AD.

With imagery data, ResNet-50 correctly classified 98.99% of the validation set and 99.34% of the training set AD by severity after 152 epochs. The multi-class classification accuracy of 98.99% achieved using image recognition and ResNet-50 are superior to others found in the literature ([11] (95%), [12] (96.5%), [16] (87%), and [18] (97%)) with the exception of another very small study in [17] (100% 10-fold CV), and such results support the notion that ML methods might be used as the first screening for diseases such as AD. In all cases, these studies consisted of smaller samples. Such results support the notion that ML methods might be used as the first screening for diseases such as AD.

Another interesting finding was that while image methods may support radiologists’ processes, non-imagery methods were reasonable at classifying the present of AD during screening. The area under the curve in eight of the 10 folds was 96% or better. MMSE and age were powerful screening tools for AD. Coupling these tools with ML imagery analysis to support radiologists and physicians may help to diagnose earlier. An MRI generated for any reason might be automatically screened as part of a best-practice policy.

The ResNet-50 multi-class classification itself is an improvement over previous studies such as [12,13] that used cross-validation (which exposes the validation set to the same pre-processing as the training set thus inducing bias) and binary classification. Further, it demonstrates that ResNet-50 can provide excellent predictions when trained and applied to Alzheimer’s MRI imagery. The study team saved the trained weights for use in additional medical image classification and is providing the code for replication.

There were several limitations of this study. First, only 51 slices of each patient’s MRI were used in the imagery analysis. Doing so might have eliminated slices that were helpful in diagnosing AD. Second, the actual number of individuals in the study, 416, was small (although the number of images was certainly sufficient). This limits the generalizability of the findings for both the GBM and ResNet models, as they would need re-training on an additional dataset. This retraining is not problematic, but slightly different classification accuracies would be likely.

Future work will investigate the classification capability of the pre-trained ResNet-50 on some additional datasets including ADNI and will extend to time series (longitudinal) MRIs. Further, some modifications of ResNet, implementation of other network schemes, and early identification based on longitudinal analysis will be explored.

What is noteworthy here is that the analysis with and without imagery resulted in reasonable classification accuracy. The implication here was that AD might be detected by the MMSE and the associated socio-demographics of the patient early on without imagery evaluation, potentially reducing costs. Further, feeding information already collected into ML algorithms might help identify patients at risk. In addition, application of ML image recognition to MRIs will successfully code and classify AD for confirmation by providers with high accuracy, even if a patient’s intended visit was not for AD. ML algorithms may flag these images automatically for review.

## Figures and Tables

**Table 1:** Descriptive statistics for dementia by age and gender (adopted from Open Access Series of Imaging Studies—OASIS, 2018).

		Non-Demented				Demented				
Age	*N*	*n*	Mean	Male	Female	*n*	Mean	Male	Female	CDR 0.5/1/2
<20	19	19	18.53	10	9	0	0	0	0	0/0/0
[20, 30)	119	119	22.82	51	68	0	0	0	0	0/0/0
[30, 40)	16	16	33.38	11	5	0	0	0	0	0/0/0
[40, 50)	31	31	45.58	10	21	0	0	0	0	0/0/0
[50, 60)	33	33	54.36	11	22	0	0	0	0	0/0/0
[60, 70)	40	25	64.88	7	18	15	66.13	6	9	12/3/0
[70, 80)	83	35	73.37	10	25	48	74.42	20	28	32/15/1
[80, 90)	62	30	84.07	8	22	32	82.88	13	19	22/9/1
[90, 100)	13	8	91.00	1	7	5	92.00	2	3	4/1/0
Total	416	316	n/a	119	197	100	n/a	41	59	70/28/2

CDR—Clinical Dementia Rating.

**Table 2:** Clinical dementia rating (CDR) frequency distribution by gender.

	CDR = 0, No Dementia	CDR = 0.5, Very Mild Dementia	CDR = 1, Mild Dementia	CDR = 2, Moderate Dementia
Male	119	31	9	1
Female	197	39	19	1
Total	316	70	28	2

**Table 3:** Descriptive statistics for the quantitative variables (*n* = 416).

Variable	Mean	SD	Median	Min	Max
Age	52.70	25.08	56	18	96
Mini-Mental State Exam	27.50	3.13	29	14	30
eTIV (Intracranial Volume)	1480.53	158.34	1475	1123	1992
nWBV (Brain Volume)	0.79	0.06	0.8	0.64	0.89
ASF (Atlas Scaling Factor)	1.2	0.13	1.19	0.88	1.56

SD—standard deviation.

## References

[1] Mayeux R., Sano M. (1999). Treatment of Alzheimer’s disease. N. Engl. J. Med..

[2] Alzheimer’s Association Facts and Figures. https://www.alz.org/alzheimers-dementia/facts-figures.

[3] VanMeter K., Hubert R.J. (2017). Gould’s Pathophysiology for the Health Professions.

[4] Bhagwat N., Viviano J.D., Voineskos A.N., Chakravart M.M. (2018). Modeling and prediction of clinical symptom trajectories in Alzheimer’s disease using longitudinal data. PLoS Comput. Biol..

[5] Amino Brain MRI Costs. https://amino.com/blog/brain-mri-cost.

[6] Driscoll I., Troncoso J. (2011). Asymptomatic Alzheimerʼs Disease: A Prodrome or a State of Resilience?. Curr. Alzheimer Res..

[7] Rubin E.H., Storandt M., Miller J.P., Kinscherf D.A., Grant E.A., Morris J.C., Berg L. (1998). A prospective study of cognitive function and onset of dementia in cognitively healthy elders. Arch. Neurol..

[8] Aljondi R., Szoeke C., Steward C. (2018). A decade of changes in brain volume and cognition. Brain Imaging Behav..

[9] Huang L., Jin Y., Gao Y., Thung K.H., Shen D. (2016). Longitudinal clinical score prediction in Alzheimer’s disease with soft-split sparse regression based random forest. Neurobiol. Aging.

[10] Small B., Backman L. (2007). Longitudinal trajectories of cognitive change in preclinical Alzheimer’s disease: A growth mixture modeling analysis. Cortex.

[11] Zhang Y., Dong Z., Phillips P., Wang S., Ji G., Yang J., Yuan T. (2015). Detection of subjects and brain regions related to Alzheimer’s disease using 3D MRI scans based on Eigenbrain and machine learning. Front. Comput. Neurosci..

[12] Zhang Y., Wang S., Phillips P., Yang J., Yuan T. (2016). Three-Dimensional Eigenbrain for the Detection of Subjects and Brain Regions Related with Alzheimer’s Disease. J. Alzheimer’s Dis..

[13] Bansal D., Chhikara R., Khanna K., Gupta P. (2018). Comparative Analysis of Various Machine Learning Algorithms for Detecting Dementia. Procedia Comput. Sci..

[14] Bhagwat N., Pipitone J., Voineskos A., Chakravarty M. (2019). An artificial neural network model for clinical score prediction in Alzheimer disease using structural neuroimaging measures. J. Psychiatry Neurosci..

[15] Collij L.E., Heeman F., Kuijer J.P.A., Sanz-Arigita E.J., Van Berckel B.N.M., Barkhof F., Wink A.M., Ossenkoppele R., Benedictus M.R., Möller C. (2016). Application of machine learning to arterial spin labeling in mild cognitive impairment and Alzheimer disease. Radiology.

[16] Lahmiri S. (2016). Image characterization by fractal descriptors in variational mode decomposition domain: Application to brain magnetic resonance. Phys. A.

[17] Lahmiri S., Schmuel A. (2019). Performance of machine learning methods applied to structural MRI and ADAS cognitive scores in diagnosing Alzheimer’s disease. Biomed. Signal Process. Control.

[18] Lahmiri S., Boukadoum M. (2014). New approach for automatic classification of Alzheimer’s disease, mild cognitive impairment and healthy brain magnetic resonance images. Healthc. Technol. Lett..

[19] Open Access Series of Imaging Studies (OASIS). http://www.oasis-brains.org/.

[20] Marcus D.S., Wang T.H., Parker J., Csernansky J.G., Morris J.C., Buckner R.L. (2007). Open Access Series of Imaging Studies (OASIS): Cross-sectional MRI data in young, middle aged, nondemented, and demented older adults. J. Cogn. Neurosci..

[21] R Core Team (2016). R: A Language and Environment for Statistical Computing.

[22] Python Software Foundation (2015). Python Language Reference.

[23] Morris J.C. (1993). The Clinical Dementia Rating (CDR): Current version and scoring rules. Neurology.

[24] Buckner R.L., Head D., Parker J., Fotenos A.F., Marcus D., Morris J.C., Snyder A.Z. (2004). A unified approach for morphometric and functional data analysis in young, old, and demented adults using automated atlas-based head size normalization: Reliability and validation against manual measurement of total intracranial volume. Neuroimage.

[25] Zujun H. (2006). A Review on MR Image Intensity Inhomogeneity Correction. Int. J. Biomed. Imaging.

[26] Talairach J., Tournoux P. (1988). Co-Planar Stereotaxic Atlas of the Human Brain.

[27] The Talairach Project. Talairach Coordinate Space. http://www.talairach.org/about.html.

[28] Cao J., Su Z., Yu L., Chang D., Li X., Ma Z. Softmax Cross Entropy Loss with Unbiased Decision Boundary for Image Classification. Proceedings of the 2018 Chinese Automation Congress (CAC).

[29] Zhang Y., Yao J. (2017). Gini objective functions for three-way classifications. Int. J. Approx. Reason..

[30] Sakata R., Ohama I., Taniguchi T. An Extension of Gradient Boosted Decision Tree Incorporating Statistical Tests. Proceedings of the 2018 IEEE International Conference on Data Mining Workshops (ICDMW).

[31] Chen L. (2019). Basic Ensemble Learning (Random Forest, AdaBoost, Gradient Boosting)- Step by Step Explaineds. https://towardsdatascience.com/basic-ensemble-learning-random-forest-adaboost-gradient-boosting-step-by-step-explained-95d49d1e2725.

[32] Tianqi C., Tong H., Michael B., Vadim K., Yuan T., Hyunsu C., Kailong C., Rory M., Ignacio C., Tianyi Z. Xgboost: Extreme Gradient Boosting. R Package Version 0.82.1. https://CRAN.R-project.org/package=xgboost.

[33] Álvarez I., Górriz J.M., Ramírez J., Salas-Gonzalez D., López M., Segovia F., Puntonet C.G. (2009). Alzheimer’s diagnosis using Eigenbrains and support vector machines. Electron. Lett..

[34] Poli R., Citi L., Salvaris M., Cinel C., Sepulveda F. (2010). Eigenbrains: The free vibrational modes of the brain as a new representation for EEG. Conf. Proc. IEEE Eng. Med. Biol. Soc..

[35] Francois C. (2015). Keras Tensorflow.

[36] He K., Zhang X., Ren S., Sun J. Deep Residual Learning for Image Recognition. Proceedings of the 2016 IEEE Conference on Computer Vision and Pattern Recognition (CVPR).

[37] Fung V. (2017). An Overview of ResNet and its Variants. https://towardsdatascience.com/an-overview-of-resnet-and-its-variants-5281e2f56035.

[38] Qiu A., Younes L., Miller M.I. (2012). Principal Component Based Diffeomorphic Surface Mapping. IEEE Trans. Med. Imaging.

